# Integrating Behavioral Health Into Monitoring and Surveillance During Public Health Emergencies: Challenges and Opportunities

**DOI:** 10.1017/dmp.2024.127

**Published:** 2024-09-18

**Authors:** Laura J. Faherty, Sara J. Vagi, Mary Leinhos, Robin E. Soler, Joie D. Acosta

**Affiliations:** 1 RAND Corporation, Boston, MA, USA; 2 Maine Medical Center, Portland, ME, USA; 3 Tufts University School of Medicine, Medford and Somerville, MA, USA; 4 Office of Readiness and Response, Centers for Disease Control and Prevention, Atlanta, GA, USA; 5 US Public Health Service Commissioned Corps, Bethesda, MD, USA; 6 RAND Corporation, Santa Monica, CA, USA

**Keywords:** emergency, behavioral health, mental health, public health, surveillance

## Abstract

**Objective:**

Limited guidance exists for public health agencies to use existing data sources to conduct monitoring and surveillance of behavioral health (BH) in the context of public health emergencies (PHEs).

**Methods:**

We conducted a literature review and environmental scan to identify existing data sources, indicators, and analytic methods that could be used for BH surveillance in PHEs. We conducted exploratory analyses and interviews with public health agencies to examine the utility of a subset of these data sources for BH surveillance in the PHE context.

**Results:**

Our comprehensive search revealed no existing dedicated surveillance systems to monitor BH in the context of PHEs. However, there are a few data sources designed for other purposes that public health agencies could use to conduct BH surveillance at the substate level. Some of these sources contain lagging indicators of BH impacts of PHEs. Most do not consistently collect the sociodemographic data needed to explore PHEs’ inequitable impacts on subpopulations, including at the intersection of race, gender, and age.

**Conclusions:**

Public health agencies have opportunities to strengthen BH surveillance in PHEs and build partnerships to act based on timely, geographically granular existing data.

Public health emergencies (PHEs, which are events, whether natural or human-made, that pose a risk to health—for example, hurricanes, pandemics, and terrorist attacks) can negatively impact behavioral health (BH). For some individuals, BH impacts, defined as new or worsening mental health conditions or substance use disorders, can be severe and long-lasting.^1^ After a PHE, approximately 1 in 4 adults will develop clinically significant posttraumatic stress syndrome and anxiety, and about 1 in 5 will develop depressive symptoms.^2^ Over time, symptoms of post-traumatic stress syndrome typically decrease, but depression and anxiety can persist. Both the direct impacts of the COVID-19 pandemic and the necessary public health measures to control its spread could have exacerbated the need for BH services. For instance, depression symptoms in children and adolescents increased during the pandemic, particularly among girls,^3^ and drug overdose mortality was higher in 2020 than in previous years.^4^

Currently, no dedicated surveillance systems exist to monitor BH during and after PHEs.^5^ Even outside of the PHE context, in lieu of having a comprehensive surveillance system for BH, public health officials rely on a patchwork of surveys and surveillance systems^6^ that have limitations, including long delays between data collection and release, nonrepresentative sampling, and lack of validated BH measures.^5,7^

With improved monitoring and surveillance of BH precursors and impacts, public health officials can understand BH trends and better anticipate potential impacts of PHEs, identify acute increases in BH needs, evaluate the effectiveness of BH interventions, position resources to prevent adverse BH impacts from future PHEs, and begin to address long-standing BH inequities that may emerge in PHEs.

To plan for, respond to, and recover from PHEs, public health agencies and their BH partners need timely local data on BH needs to develop tailored public health interventions. However, little is known about the strengths and limitations of existing data sources, indicators of population-level BH, and methodological approaches for conducting BH surveillance before, during, and after PHEs. Furthermore, there is limited guidance on how public health agencies can use BH surveillance data to better prepare for and respond to PHEs.

To fill these gaps, we conducted a mixed-methods study to answer 2 primary research questions (RQs):

RQ 1: What indicators, data sources, and analytic approaches could be used for BH surveillance in the PHE context?

RQ 1.1. What types of BH indicators are needed for surveillance in the PHE context?

RQ 1.2. What existing data sources contain these BH indicators, are available at the substate level, and are updated frequently enough to be useful in the PHE context?

RQ 1.3. How can public health agencies access and analyze these data, and what are the potential strengths and limitations of the different data sources?

RQ 2: How could better BH surveillance before, during, and after PHEs support decision-making and public health action?

RQ 2.1. How can these data be used to inform public health decision and action?

RQ 2.2. What do public health officials perceive as best ways to use BH analyses to inform decisions and public health action?

## Methods

We conducted a literature review and environmental scan to identify data sources, indicators, and analytic approaches that could be used for BH surveillance in the PHE context. We then conducted exploratory analyses and interviews with public health officials to examine the utility of a subset of these data sources for BH surveillance in PHEs (Figure S.1). The corresponding author’s Human Subjects Review Committee exempted this study from further review (2019–0920-AM03).

### RQ 1: Identifying BH Indicators, Existing Data Sources, and Analytic Approaches

#### RQ 1.1: Review of systematic reviews and meta-analyses to identify indicators

To develop a conceptual model of potential types of BH indicators for surveillance in the PHE context, we conducted a keyword search of the PubMed, PsycINFO, and Web of Science databases (Supplemental Online Appendix Table S.1). To focus on the most current information available within a rapidly changing landscape, we limited the search to English-only systematic reviews and meta-analyses from the prior 5 years: 2014 to November 2019 (the date the search was conducted). We screened resulting references for relevance and systematically abstracted information from included articles.

#### RQ 1.2 and 1.3: Environmental scan to identify data sources and analytic methods

We then performed an environmental scan consisting of a second literature review and interviews with representatives of organizations that collect or aggregate data relevant to BH (e.g., Poison Control Centers, the National Retail Data Monitor), representatives from public health agencies, and experts in public health and BH. The purpose was to identify data sources that contained relevant BH indicators and analytic methods that have been used to examine those indicators.

##### Literature search.

We searched the peer-reviewed literature published in English from 2010 to 2020 using PubMed and PsycINFO and the search terms in Supplemental Online Appendix Table S.2. We also conducted searches for grey literature on Google Scholar for selected data sources using search terms tailored to each source (Supplemental Online Appendix Table S.2). After screening articles for relevance, we again systematically abstracted information from included articles.

Findings from the search were used to narrow down the list of data sources to a subset that was assessed to be promising for BH surveillance in the PHE context, defined as meeting the following 3 criteria: available (1) at the sub-state level, (2) more frequently than once a year, and (3) for most jurisdictions in the United States.

##### Informational interviews with organizations that collect or aggregate data relevant to BH.

We held 21 informational interviews lasting 45 to 60 minutes with representatives of organizations that collect or aggregate data relevant to BH, focusing on the subset of promising data sources. We identified these individuals through targeted web searches and chain-referral sampling. After obtaining verbal consent, we used a semi-structured discussion guide (Supplement 1) to assess how public health agencies could access and analyze these data, and strengths and limitations of the data sources. We analyzed our detailed notes using a qualitative descriptive approach.

##### In-depth interviews with public health agencies and experts.

We conducted 11 interviews lasting 45 to 60 minutes with state and local public health agencies and experts in public health and BH, selected for their experience with these data sources and analytic approaches for BH surveillance. After obtaining verbal consent, we used a semi-structured discussion guide (Supplement 1) that covered challenges with BH surveillance, potential data sources, and analytic methods. Interviews were audio-recorded and transcribed. Transcripts were coded in Dedoose for common themes.

Findings from both the informational and in-depth interviews informed the design of our exploratory analyses (RQ 2.1).

### RQ 2: Examining the Utility of the Promising Data Sources

#### RQ 2.1: Exploratory analyses

To answer RQ 2.1, we conducted exploratory analyses of the promising data sources. We obtained datasets for locations that had experienced one or more PHEs, had sufficient population sizes for robust analyses, and were in different regions of the country. Some of these sources were publicly available, whereas others required data requests and data use agreements. For each source, we followed a structured process to examine the utility of the data source for BH surveillance in PHEs (Supplemental Online Appendix S.3). We documented our qualitative assessments at each step, which we integrated with interview findings described below (RQ 2.2) to arrive at our final assessment of the sources. Each source required slightly different analytic methods, which are presented elsewhere.^8–11^

#### RQ 2.2: Interviews with public health agencies and data experts

We conducted 60-minute in-depth interviews lasting 45 to 60 minutes with 37 state, tribal, and local public health representatives; subject matter experts in public health and BH; and data source experts. We identified interview participants through chain-referral recommendations and the literature search for RQs 1.2 and 1.3. After obtaining verbal consent, we used a semi-structured discussion guide (Supplement 2) to gather input on how findings from the exploratory analyses (RQ 2.1) could inform public health action, focusing on the utility of these findings for BH surveillance in PHEs. Interviews were audio-recorded and transcribed, and transcripts were coded in Dedoose for common themes.

## Results

### RQ 1: Identifying BH Indicators, Existing Data Sources, and Analytic Approaches

#### RQ 1.1: A conceptual model of BH indicators

**Figure 1.**
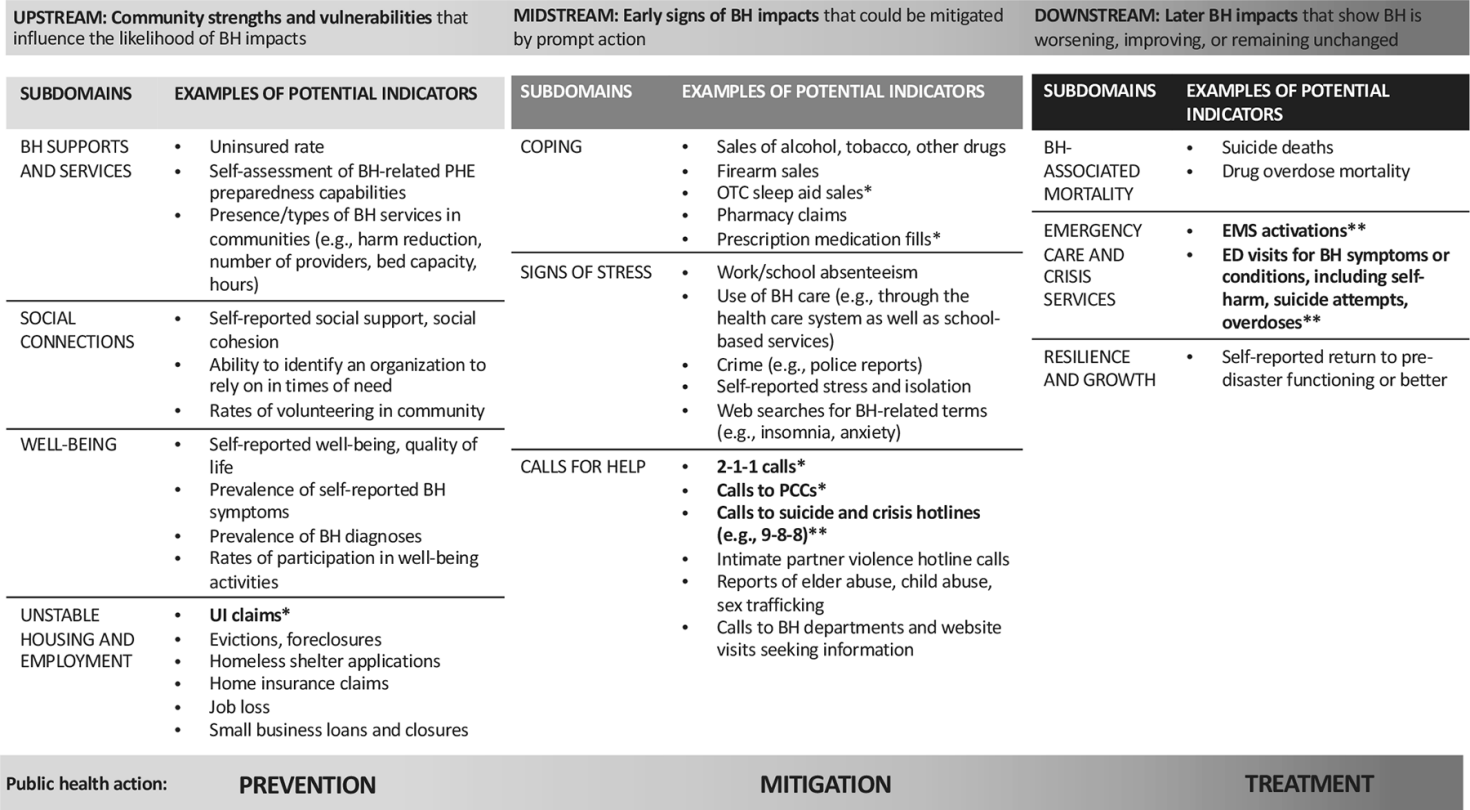
Conceptual framework of indicators of community strengths and vulnerabilities and behavioral health impacts of public health emergencies.^1,2^ *Promising data sources for which we conducted exploratory analyses **Promising data sources that we assessed through the environmental scan and in-depth interviews but for which we did not conduct exploratory analyses ^1^ Abbreviations: BH: behavioral health; PHE: public health emergency; UI: unemployment insurance; OTC: over-the-counter; PCC: Poison Control Centers; EMS: emergency medical services; ED: emergency department ^2^ Promising data sources that could be repurposed for BH surveillance during and after PHEs are bolded

Through the literature review and environmental scan, we identified approximately 40 types of BH indicators that could be surveilled in the PHE context. We developed a conceptual model that groups these indicators into 3 categories (Figure 1):

- Upstream indicators: community strengths, vulnerabilities, and other social determinants of BH that may influence the likelihood that a future PHE will impact population-level BH. These include risk and protective factors such as unemployment or job loss, housing instability, and social cohesion, several of which have been linked to BH.^12–14^
- Midstream indicators: early signs of BH impacts after the PHE has occurred for which there is still time to intervene and mitigate their effects. These include “calls for help” when BH is declining, such as to poison control centers (PCCs) for intentional ingestions, or measures of coping strategies such as taking over-the-counter sleep aids for insomnia associated with stress or depression.^15^
- Downstream indicators: measures of a PHE’s later impacts on BH. These include emergency department (ED) visits for a BH crisis, calls for emergency medical services (EMS) to respond to an overdose^16^ or BH emergency, or deaths related to overdose^21^ or suicide.^17^

Not all the indicators in Figure 1 are available in the data sources discussed next. However, they are included for completeness, as public health agencies may have access to local data sources that are not available in other jurisdictions.

#### RQ 1.2: Existing data sources

The literature review, environmental scan, and formative interviews yielded 27 data sources containing one or more of the BH indicators we identified but no single dedicated data source for monitoring BH in the PHE context.

Applying the 3 criteria described previously to those 27 sources (i.e., available (1) at the substate level, (2) more frequently than once a year, and (3) for most jurisdictions in the United States) yielded 8 data sources that were considered promising for use in BH surveillance during and after PHEs:

- unemployment insurance claims
- calls to 2–1-1 centers
- calls to poison control centers for intentional ingestions
- over-the-counter medication sales of sleep aids
- pharmacy data on psychotropic medication fills
- EMS activations
- ED visits, and
- web searches for BH-related terms.

These sources contained indicators in the upstream, midstream, and downstream categories.

Two of these sources, EMS activations and ED visits, had national dashboards or well-established national surveillance programs, so we did not obtain these data for primary data analysis and instead used in-depth interviews (RQ 2.2) to solicit examples of how they have been used for BH surveillance in PHEs.

Next, web search data (i.e., aggregated through the Google Trends website) was excluded from the list of promising data sources after our exploratory analyses failed to detect a BH signal in these data, and we found that the data source did not have sufficient spatiotemporal granularity to be useful to state and local public health officials.

Finally, based on the in-depth interviews, we added calls to the 9–8-8 Suicide and Crisis Lifeline to the above list of promising data sources, bringing the total back to 8. The 9–8-8 dialing code launched in July 2022 and therefore did not “go live” in time for us to obtain data for analysis.

Table 1 summarizes how to access the data sources, their spatio-temporal granularity, and their strengths and limitations. Bolding indicates the 5 sources with which we conducted our own exploratory analyses (unemployment insurance claims, calls to 2–1-1 centers, calls to poison control centers for intentional ingestions, over-the-counter medication sales of sleep aids, and pharmacy data) (Supplemental Appendix S.3). Our assessment of EMS activations, ED visits, and calls to 9–8-8 was based on the environmental scan (RQ 1.2 and RQ 1.3) and semi-structured interviews (RQ 2.2).

Overall, existing data sources lacked data on sociodemographic factors that would be necessary to examine BH inequities. Only unemployment insurance claims and ED visit data consistently contained information on age, gender, and race of the claimant or patient. Age and gender were available in the data on poison control center (PCC) calls and prescription medication fills; however, race was not. Only gender was consistently collected from callers to 2–1-1 centers. Age, race, and gender were inconsistently included or not available in data on sleep aid sales, EMS activations, and suicide and crisis hotline calls.

Although other data sources contain BH indicators (e.g., those compiled by the Council of State and Territorial Epidemiologists through an initiative to strengthen surveillance of mental health and substance use),^18^ these indicators are not available frequently enough to be useful in the PHE context (Supplemental Online Appendix Table S.4).

#### RQ 1.3: Analytic approaches

We identified over 100 studies that described possible analytic approaches to monitor BH indicators (Table 2).^19^ Most studies used retrospective and observational repeated cross-sectional or longitudinal analytic approaches aggregating information across space (e.g., census tract) and/or time (e.g., weekly), rather than relying on prospective data collection using a counterfactual or comparison group. Analytic methods varied based on the question(s) to be explored or the indicator(s) to be monitored; the type(s) and volume of data available for analysis; the timing in relation to a PHE; and the available resources, capacity, and technical proficiency that the PH agency and its partners bring to this work. Commonly used methods for time series analyses of BH indicators included data-smoothing techniques followed by statistical modeling such as interrupted time series analyses or ITSA, difference-in-difference, and ARIMA (i.e., autoregressive integrated moving average) modeling.

### RQ 2: Examining the Utility of Existing Data Sources

#### RQ 2.1: Uses of these data sources to inform public health decisions and action

Table 3 summarizes methods and key findings from our exploratory analyses using 5 of the promising data sources in the context of PHEs. Full details of these analyses can be found elsewhere.^19^ Across the multiple locations we examined and different PHE types, we found that changes in indicators of BH risks and impacts of PHEs can be detected in these data sources, suggesting the utility of these data to inform public health decision-making.

Overall, we observed anomalies in the indicators we examined across most of the promising data sources for the start of the COVID-19 pandemic, but anomalies in these indicators around more acute, localized PHEs were more difficult to detect. For instance,

- in Los Angeles County, unemployment insurance claims peaked just after California’s business closure mandate in April 2020 and again in September 2020, coinciding with severe wildfires. In June 2020, SSRI fills began to increase.
- in Los Angeles County, we detected 4 periods of anomalous activity in over-the-counter sleep aid sales, coinciding with the 4 large COVID-19 waves there.
- in Broward County, Florida, calls to 2–1-1 after Hurricane Irma and the pandemic declaration showed a larger percent increase over baseline for men than for women. Calls by women remained elevated for a longer period than by men after the hurricane. The opposite was found after the pandemic declaration.
- by early 2021, calls to PCCs for intentional exposures among youth in Dallas County, Texas, increased after the pandemic declaration, closing the longstanding gap for intentional exposures between youth and adults. We detected an increase in calls for intentional exposures in this county during the summer surge in COVID-19 cases but not shortly after the pandemic declaration. The winter storm of February 2021 was not associated with an increase in calls to PCCs for intentional ingestions.

#### RQ 2.2: Public health officials’ perceptions of the utility of these analyses to inform public health action

Public health agency representatives identified several public health actions that could result from timely BH surveillance efforts.

- Before a PHE: Based on surveillance of upstream BH indicators, public health agencies and their partners could develop protective interventions, advocate for policies, and allocate resources to mitigate BH risks.
- During and after a PHE: With better surveillance of mid- and downstream BH indicators, public health agencies and their partners could alert emergency management services, BH, and other health-care providers to prepare for possible surges in demand for services and could advocate for resources to respond to those needs.

To increase their confidence in potential surveillance signals and to inform public health action, public health practitioners reported the need to compare findings across multiple indicators. Even among jurisdictions that have explored the integration of various data streams into their BH surveillance efforts, such as Washington State^20^ and North Carolina,^21^ interviewees noted some persistent challenges. More research is needed to (1) understand to what extent anomalies detected in these data sources correlate with “true” changes in population prevalence of BH needs and (2) use a signal in one data source (e.g., unemployment claims) to predict when other downstream signals might emerge (e.g., increases in ED visits for BH).

## Discussion

Our study did not identify any existing dedicated surveillance systems to monitor BH in the context of PHEs.^5^ However, there are a limited number of data sources that public health agencies in the United States could repurpose for timely BH surveillance at the substate level. We were able to detect “signals” of population-level BH shifts in these data sources, even though these data were not originally collected for this purpose. The magnitude and duration of anomalies in the BH indicators varied by PHE, age, and gender. Consistent with our conceptual model, changes in time series of BH indicators occurred at different time points in the PHE context, with upstream indicators peaking first followed by mid- and downstream indicators.

Existing data sources were limited and primarily allowed for monitoring of lagging, rather than leading, BH indicators. Aside from unemployment claims, the data sources we identified had limited information about structural inequities and well-being, which are key factors related to BH. Only a few data sources consistently collect the sociodemographic data needed to examine PHEs’ inequitable impacts on subpopulations, including at the intersection of race, gender, and age.

Additional data sources with the spatiotemporal granularity required to be useful in the PHE context are needed to more comprehensively monitor upstream social determinants of health that influence BH in the short and longer term. Efforts to modernize and transform public health data systems in the United States^22,23^ are underway to improve the consistency and completeness of data on sociodemographic factors to allow for subgroup analyses and better target public health interventions. These initiatives will benefit from close coordination among public health and BH stakeholders at federal, state, and local levels to clearly define roles and responsibilities around data collection, analysis, reporting, and action.

Emerging technologies and advanced analytic methods also offer opportunities for continued progress in strengthening BH surveillance, during both PHEs and routine times. For example, wastewater surveillance is increasingly being applied in jurisdictions across the country to monitor trends in levels of opioids in wastewater.^24^ As these emerging technologies come into more widespread use, they may provide important complementary data for more integrated, timely, equitable, and actionable BH surveillance during PHEs.^25^

To use these data for public health action, additional capabilities and partnerships are necessary. Platforms for aggregating and visualizing BH data are advancing. For instance, the National Emergency Medical Services Information System has added BH indicators to their dashboard,^26^ and the National Retail Data Monitor has added over-the-counter sleep aids to its data categories. However, partnerships that include key local organizations from the BH and public health systems are necessary for data interpretation and dissemination.

Experts noted the importance of triangulating among different data sources. However, even after comparing findings across different data sources and using statistical methods to test for significant shifts in BH indicators, decision-makers may face uncertainty around the best course of action to take given the findings. Public health officials will need to acknowledge the gaps in existing data when co-interpreting findings with the affected populations and conveying results and recommendations to different audiences.

### Limitations

This work has some limitations. First, the literature review, although rigorous in its approach, was not designed to identify all relevant articles on this broad topic, so it is possible that there are other BH indicators that could be considered for surveillance in the PHE context. Second, the exploratory analyses are intended to be preliminary examinations of the promising data sources we identified. They were conducted retrospectively, using selected past PHEs, including hurricanes, wildfires, a severe winter storm, and the onset of the COVID-19 pandemic, rather than as the PHEs unfolded, which would have allowed us to draw conclusions about their utility for BH surveillance in real time. In addition, the findings from these analyses may not be generalizable to other PHEs and to other locations.

## Conclusions

Effectively monitoring and addressing BH needs, both during a PHE and in routine times, depends on timely BH surveillance data with sufficient geographic granularity to inform just-in-time decision-making. Although our study did not identify a dedicated surveillance system to monitor BH in the context of PHEs, several existing data sources could be repurposed by public health agencies to strengthen their BH surveillance efforts in the PHE context.

### Implications for Public Health Practice

In the near term, while data modernization efforts are underway, public health officials could use the data sources identified in this study, supported by a tool kit with instructions on how to access and analyze each of them.^19^ These data modernization efforts are an opportunity to ensure that BH indicators are integrated into these changing systems so that they could be monitored and surveilled to inform the BH response to PHEs. Public health officials also could focus on leveraging existing partnerships between BH and public health systems or forming new ones, and they could access existing data aggregation platforms such as the National Emergency Medical Services Information System that are increasingly incorporating BH indicators into their dashboards. Public health workforce development efforts could focus on building data science, analytic, modeling, and informatics skills that specifically apply to BH.

Over the longer term, efforts to modernize public health data systems in the United States could explicitly include strategies to update core data and surveillance infrastructure for BH so that public health officials can monitor emerging BH impacts of PHEs, detect and intervene earlier on population-level BH concerns during and after PHEs, and access technology that reduces the collection and reporting burden for BH indicators.

With continued efforts to understand BH risks, needs, and impacts for the overall population as well as key subpopulations, public health action to combat the current BH crisis can be more tailored, proactive, and equitable.

## Supplementary Material

Supplement 2

Supplement 1

## Figures and Tables

**Table 1.** Summary of the most promising data sources for behavioral health surveillance in the context of public health emergencies^a^

Data source/indicator	How public health agencies may access the data	Spatio-temporal granularity	Strengths	Limitations
Upstream indicator				
Unemployment insurance claims	Publicly available from US. Department of Labor (DOL) and state labor and workforce agencies^28^	State/week from US DOL County/week from some state agencies	• Data collection and dissemination is legislatively mandated, and thus is consistent, stable, and publicly available. • Data are released on a timely basis. • COVID-19 response efforts have prompted many states to create publicly accessible dashboards showing these data.	• Data representativeness and validity may be limited because not all workers are eligible or apply for unemployment insurance. • Compared to all unemployed persons, claimants are more likely to be White, male, and union members (however, COVID-19 may have increased public awareness about the availability of this insurance and the application process, such that claims data may now be more representative of unemployed adults). • Changes in the sociodemographic distribution of unemployment claims data following a PHE is likely a combination of differential impacts of the event on certain sociodemographic groups and changes in claiming behavior.
Midstream indicators				
Calls to 211 agencies/call centers	Data sharing arrangement with local 211 call center Publicly available data on *211 counts* website^29^	Zip code/time of day	• Available for most of the country. • *211 counts* provides an aggregated dashboard to access local data. • Calls are tagged with a call time and zip code.	• Certain demographic variables are often missing. • Call need/call type is not always documented and while a standard taxonomy of call need exists, not all centers use it, making comparisons across call centers challenging. • Systematic missingness and higher likelihood of misclassification in call need occur in the PHE context because of increases in call volume. • Local arrangements among 211 agencies and 988 suicide and crisis lifelines may influence patterns in 211 calls for BH reasons. • An increase in 211 call volume may be the result of public awareness campaigns rather than true increases in need.
Calls to Poison Control Centers (PCC) classified as “intentional” ingestions	Data sharing agreement with local PCC to access the National Poison Data System (NPDS) portal^30^ Purchase NPDS data through the American Academy of Poison Control Centers	Zip code/time of day	• Data are timely and complete. • The data structure is consistent across states and zip codes, allowing for comparisons among jurisdictions. • Custom datasets can be requested from NPDS.	• Data are not population–representative and are based on self–report. • People seeking information about a potential exposure through the PCC website rather than calling are not captured in these data. • Caller race/ethnicity is not available. • Intentional ingestions may not be related to suspected suicide and instead represent misuse of a substance without the goal of self-harm (e.g., ingesting bleach or chloroquine phosphate due to misinformation about COVID-19 treatments). • Depending on arrangements with PCCs, there may be a cost to obtain these data, and costs are higher for more granular data. • It may take several weeks to receive requested data from AAPCC.
Pharmacy data/fills of psychotropic medications (e.g., antidepressants)	Varies by data source (e.g., we purchased IQVIA Xponent data)^31^	Varies by data source	• Given the time required to seek mental health care, receive a prescription, then fill it at a pharmacy, this indicator can be useful for monitoring ongoing BH impacts that do not self-resolve. • The metric of newfill rates for medications can be easily calculated and interpreted. • IQVIA products have spatio temporal granularity, capture all payer types, and can be delivered on a frequent and timely basis, depending on your agreement with IQVIA.	• Changes in fills may be the result of changes in mental health need or changes in access to care (e.g., “met need” for depression treatment). • The reported rate of new prescriptions may include current users of a particular medication who ran out of refills, switched pharmacies, or switched prescribers. • IQVIA data do not include a variable for race or ethnicity, and other sources of pharmacy data may have different limitations, such as less spatio–temporal granularity—or, in the case of pharmacy claims data, greater administrative and analytic burden. • IQVIA data are not publicly available and must be purchased.
OTC sleep aids	Through a subscription to the National Retail Data Monitor (NRDM)^32^	Zip code/day	• It is clear what measured quantities represent. • NRDM data are available within days. • Compared with similar datasets, the NRDM is available at low cost. • The NRDM has almost two decades of data availability; the platform is accessible around-the-clock, with minimal downtime. • The NRDM has a long history of use by several PH agencies. • The user interface and in-built analytics streamline analyses.	• Outside factors, such as advertising or promotions, can alter purchasing behavior. • Purchasing behavior varies throughout the year. To help account for this, the NRDM reports normalized sales that are calculated by dividing each category’s sales by the total sales across all categories. • The zip codes for purchases reflect where the purchase occurred, not the customer address. • Those who shop with local businesses or online may not be reflected. • Not all relevant products are tracked (e.g., cannabinoids for stress coping). • If there is no stock in the stores (e.g., due to panic buying), this censors the signal. • A lack of consumer information limits the ability to analyze inequities or the impact of PHEs on the BH of specific groups.
Calls to 988 Suicide and Crisis Hotlines	Through data-sharing agreements with the national implementer of 988 hotline and/or local 988 call centers^33^	Unable to fully assess	Unable to fully assess.	Unable to fully assess.
Downstream indicators				
EMS activations	Through NEMSIS^26^ or through data-sharing agreements with state or local EMS agencies	Health-care facility/day	• High spatio temporal granularity. • Some demographic characteristics are collected. • Data elements at state and national levels are standardized, cleaned, and quality-checked before release. • Data are free and accessible. • These data can be used to monitor BH problems that would not be captured in other data, such as ED visits.	• EMS coding of the reason for the activation may not be consistent with the final diagnosis. EMS data rely on ICD-10 codes as documented by EMS. Responders may not be able to ascertain whether the event was intentional, particularly if the patient is not able to communicate. • Users of EMS data must take into account seasonal trends (e.g., compare with 30 days prior to disaster or with the same month in the prior year) as well as changes in data sources and sample size that can affect trends and interpretation.
ED visits	Through CDC’s National Syndrome Surveillance Program^34^	Health-care facility/day	• Data are very timely; most are reported within 24 hours. • High spatio temporal granularity. • Inclusion of patient demographics including race/ethnicity. • Free and accessible to public health practitioners. • Multiple resources available on an online Knowledge Repository and through a Community of Practice. • Users can integrate other sources of data into the platform.	• Not fully representative; lack federal hospitals. • Patient encounter data rely on capacity of health facilities to report them, and if a hospital experiences a surge of patients or a power outage, reporting may be incomplete. • Definitional issues and interpretation of syndromes, particularly around BH; however, the NSSP has created standardized mental health condition definitions. • Important to account for overall ED visits when examining trends and consider relative counts or percentage of overall ED visits that are for a BH condition. • Potential differences in syndrome definitions, measures of interest, and reporting stems from the jurisdiction-dependent nature of the NSSP’s design and implementation as well as variable integration of additional data sources.

a Adapted from Acosta et al. ^19^

Abbreviations: BH, behavioral health; CDC, Center for Disease Control and Prevention; ED, emergency department; EMS: emergency medical services; ICD-10, International Classification of Diseases, 10th Revision; NEMSIS, National Emergency Medical Services Information System; NPDS, National Poison Data System; NRDM, National Retail Data Monitor; NSSP, National Syndromic Surveillance Program; OTC, over-the-counter; PCC, Poison Control Centers; PHE, public health emergency; UI, unemployment insurance.

**Table 2.** Illustrative publications that use the promising data sources to monitor behavioral health^a^

Data source/indicator	Publications	Analytic methods used
Unemployment insurance claims^b^	• Bidargaddi N, Bastiampillai T, Schrader G, et al. Changes in monthly unemployment rates may predict changes in the number of psychiatric presentations to emergency services in South Australia. *BMC Emergency Medicine.* 2015;15. https://www.ncbi.nlm.nih.gov/pubmed/26205556 • van der Velden PG, Muffels RJA, Peijen R, et al. Wages and employment security following a major disaster: a 17–year population-based longitudinal comparative study. *PLoS One.* 2019;14(3). https://www.ncbi.nlm.nih.gov/pmc/articles/PMC6440641/ • Xiao Y, Nilawar U. Winners and losers: analyzing post-disaster spatial economic demand shift. *Disasters.* 2013;37(4). https://onlinelibrary.comwiley.com/doi/pdf/10.1111/disa.12025	Simple correlations, time series analysis, ARIMA, fixed effects panel regression, ordinary least squares regression, spatial panel modeling, structural break-point analysis
Calls to 211 agencies/call centers	• Bame SI, Parker K, Lee JY, et al. Monitoring unmet needs: using 2–1–1 during natural disasters. *American Journal of Preventive Medicine.* 2012;43(6, Supp. 5). https://pubmed.ncbi.nlm.nih.gov/23157762/ • Eddens KS, Alcaraz KI, Kreuter MW, et al. A 2–1–1 research collaboration: participant accrual and service quality indicators. *American Journal of Preventive Medicine.* 2012;43(60). https://www.sciencedirect.com/science/article/abs/pii/S0749379712006290?via%3Dihub • Sharareh N, Hess R, Wan N, et al. Incorporation of information–seeking behavior into food insecurity research. *American Journal of Preventive Medicine.* 2020;58(6). https://www.sciencedirect.com/science/article/abs/pii/S0749379720300441	Descriptive univariate analysis, *t*–tests, regression modeling, spatial mapping and analyses (e.g., optimized outlier analysis)
Calls to Poison Control Centers (PCC) classified as “intentional” ingestions	• Klein KR, Herzog P, Smolinske S, et al. Demand for poison control center services “surged” during the 2003 blackout. *Clinical Toxicology.* 2007;45(3). https://www.ncbi.nlm.nih.gov/pubmed/17453875 • Nathan AR, Olson KR, Everson GW, et al. Effects of a major earthquake on calls to regional poison control centers. *Western Journal of Medicine.* 1992;156(3). https://www.ncbi.nlm.nih.gov/pubmed/1595244	Descriptive univariate analysis before and after the PHE; applied statistical detection algorithms to time series data to detect an aberration from the trend; change analysis before and after an event
Pharmacy data/fills of psychotropic medications (e.g., antidepressants)	• DiMaggio C, Galea S, Madrid PA. Population psychiatric medication prescription rates following a terrorist attack. *Prehospital and Disaster Medicine* 2007;22(6). https://pubmed.ncbi.nlm.nih.gov/18709935/ • Fassaert T, Dorn T, Spreeuwenberg PMM, et al. Prescription of benzodiazepines in general practice in the context of a man–made disaster: a longitudinal study. *European Journal of Public Health* 2007;17(6). https://pubmed.ncbi.nlm.nih.gov/17412715/ • Han K–M, Kim K–H, Lee M, et al. Increase in the prescription rate of antidepressants after the Sewol Ferry disaster in Ansan, South Korea. *Journal of Affective Disorders.* 2017;219. https://www.ncbi.nlm.nih.gov/pubmed/28505500	Multivariable logistic regression, regression–based quasi–experimental approaches, such as difference–in–difference analyses and interrupted time series analyses
OTC sleep aids	• Das D, Metzger K, Heffernan R, et al. Monitoring over–the–counter medication sales for early detection of disease outbreaks—New York City. *Morbidity and Mortality Weekly Report.* 2005;54. https://www.ncbi.nlm.nih.gov/pubmed/16177692 • Hogan WR, Tsui F–C, Ivanov O, et al. Detection of pediatric respiratory and diarrheal outbreaks from sales of over–the–counter electrolyte products. *Journal of the American Medical Informatics Association.* 2003;10(6). https://www.ncbi.nlm.nih.gov/pmc/articles/PMC264433/	Time series analyses with anomaly detection algorithms; cyclical linear regression modeling; Poisson regression analysis; cross–correlations
Calls/Texts to Suicide and Crisis Hotlines	• Sugg MM, Dixon PG, Runkle JD. Crisis support-seeking behavior temperature in the United States: Is there an association in young adults and adolescents?” *Science of the Total Environment.* 2019;669. https://www.ncbi.nlm.nih.gov/pubmed/30884264 • Thompson LK, Michael KD, Runkle J, et al. Crisis text line use following the release of Netflix series 13 reasons why season 1: time–series analysis of help–seeking behavior in youth. *Preventive Medicine Reports.* 2019;14. https://www.sciencedirect.com/science/article/pii/S2211335519300154 • Thompson LK, Sugg MM, Runkle JR. Adolescents in crisis: a geographic exploration of help–seeking behavior using data from crisis text line. *Social Science and Medicine.* 2018;215. https://www.sciencedirect.com/science/article/abs/pii/S0277953618304490?via%3Dihub	ARIMA, interrupted time series analysis, distributed lag nonlinear regression modeling; spatial error regression modeling
EMS activations	• Glober N, Mohler G, Huynh P, et al. Impact of COVID–19 pandemic on drug overdoses in Indianapolis. *Journal of Urban Health.* 2020;97(6). https://www.ncbi.nlm.nih.gov/pmc/articles/PMC7529089/ • Khatri UG, Pizzicato LN, Viner K, et al. Racial/ethnic disparities in unintentional fatal and nonfatal emergency medical services–attended opioid overdoses during the COVID–19 pandemic in Philadelphia. *JAMA Network Open.* 2021;4(1). https://jamanetwork.com/journals/jamanetworkopen/fullarticle/2775360 • Lindstrom HA, Clemency BM, Snyder R, et al. Prehospital naloxone administration as a public health surveillance tool: a retrospective validation study. *Prehospital and Disaster Medicine.* 2015;30(4). https://pubmed.ncbi.nlm.nih.gov/26061280/ • Slavova S, Rock P, Bush HM, et al. Signal of increased opioid overdose during COVID–19 from emergency medical services data. *Drug and Alcohol Dependence* 2020;214. https://www.ncbi.nlm.nih.gov/pmc/articles/PMC7351024/	Descriptive statistics (frequencies and proportions across periods), spatial analyses, time series analyses (including segmented regression and ARIMA models), cross–correlational analyses, bivariate analyses, and logistic regression (to predict the characteristics of patients receiving multiple naloxone administrations by EMS providers)
ED visits	• Fangtao TH, De La Cruz NL, Olson D, et al. Temporal and spatial patterns in utilization of mental health services during and after Hurricane Sandy: Emergency department and inpatient hospitalizations in New York City. *Disaster Medicine and Public Health Preparedness.* 2016;10(3). https://www.ncbi.nlm.nih.gov/pubmed/27292172 • Holland KM, Jones C, Vivolo–Kantor AM, et al. Trends in US emergency department visits for mental health, overdose, and violence outcomes before and during the COVID–19 pandemic. *JAMA Psychiatry.* 2021;78(4). https://www.ncbi.nlm.nih.gov/pubmed/33533876 • Hou W, Brutsch E, Dunn AC, et al. Using syndromic data for opioid overdose surveillance in Utah. *Online Journal of Public Health Informatics.* 2018;10(1). https://journals.uic.edu/ojs/index.php/ojphi/article/view/8988/7240 • Leeb RT, Bitsko RH, Radhakrishnan L, et al. Mental health–related emergency department visits among children aged <18 years during the COVID–19 pandemic—United States, January 1–October 17, 2020. *Morbidity and Mortality Weekly Report.* 2020;69(45). https://www.cdc.gov/mmwr/volumes/69/wr/mm6945a3.htm	Descriptive univariate and graphical approaches (e.g., trend plots), bivariate analyses (chi–squared tests, *z*–tests), and most performed some type of regression modeling (e.g., a generalized linear Poisson model for count data, generalized linear mixed effects model for longitudinal data, joinpoint regression, or ARIMA for autocorrelated data).

a For a complete list of publications identified through our environmental scan, see Acosta et al. ^19^

b The studies included for this data source differ from the others in thatthey demonstrate an association between unemployment (the indicator of interest) and behavioral health or public health emergencies.

**Table 3.** Summary of exploratory analyses of five of the most promising data sources for behavioral health surveillance in the public health emergency context

Data source/indicator	Analytic approach			Key findings
	Location, time period	PHEs during time period	Methods	
Unemployment insurance claims^11^	Los Angeles County, California Jan 2018–May 2021	COVID-19; Sept. 2020 wildfires	• Time series plot • Interrupted time series analysis (ITSA) • Difference-in-difference analysis	• ITSA: Unemployment insurance claims significantly increased (141 900; 95% CI 107 200 to 176 600) the week after the COVID-19-related business closure mandate and four weeks following the wildfires (exceeding the 95% CI) • Difference-in-difference: In the context of higher unemployment levels due to the ongoing pandemic, the peak in COVID-19 cases had a much greater (and significant) impact on weekly UI claims than the wildfires.
2–1–1 calls^9^	Broward County, Florida Sep 2016–May 2020	2017 hurricane, COVID-19	• Time series plot (of 7–day moving average) • Autoregressive integrated moving average (ARIMA) modeling with alert thresholds • ITSA	• ITSA: Both Hurricane Irma (+80.9 calls/day) and the COVID-19 pandemic declaration (+84.4calls/day) were associated with significant increases in 2–1–1 call volume. By gender, these PHEs were associated with larger absolute increases for women (+65.7 and +57.1 calls/day, respectively, compared with +15.4 and +27 calls/day for men), but larger percent increases above their baseline for men (+143% and +174% compared with +119% and +138% for women). Calls by women were elevated longer than calls by men after Hurricane Irma (5 weeks vs. 1 week, respectively), but we found the opposite after the pandemic declaration (8 vs. 21 weeks, respectively).
Poison Control Center Calls for intentional ingestions^8^	Dallas County, Texas Mar 2019–May 2021	COVID-19, severe winter storm leading to widespread power outages (“Uri”)	• Time series plot (of 7–day moving average) • ITSA	• ITSA: The summer surge in COVID-19 cases was associated with 1.9 additional intentional exposure calls/day (95% CI 0.7 to 3.1), over a baseline unadjusted mean of 6.2 calls/day (unadjusted) prior to March 11, 2020. Neither the pandemic declaration nor the winter storm was significantly associated with changes in call volume. Women made 1.2 more calls per day on average compared to men. IE calls for youth ages 0–19 years old increased after the pandemic declaration, closing the longstanding gap between adults and youth by early 2021.^8^
SSRI fills^10^	Los Angeles County, California Mar 2019–Jun 2021	COVID-19; Sept. 2020 wildfires	• Time series plot • Plot of ratio of new SSRI fills after COVID-19 to same month pre–COVID-19 • Interrupted time series analysis (ITSA)	• Time series plot of ratios: In May 2020, the ratio of post-COVID fills to pre-COVID fills (i.e., blue line) declined by more than 5%, but beginning in June 2020, the ratio consistently exceeded 1.0, indicating that the fill rate was higher than the same month pre–pandemic. For many months after the declaration of the COVID-19 national emergency, SSRI fill rates showed an increase of more than 5% compared with the same month pre–pandemic, and in March 2021 and June 2021, we observe increases of more than 10% compared with the same pre–pandemic month. • ITSA: Controlling for seasonal trends, we found a significant decrease in new SSRI fills immediately after the start of COVID-19 (*p* < 0.01) and a significantly greater slope of the trend line after the onset of COVID-19 compared with the pre–COVID-19 slope (*p* < 0.05). The outcome levels and slopes from before and after the wildfires were not significantly different from each other (*p* > 0.05).
Over–the–counter sleep aid sales	Los Angeles County, California Jan 2017–Jun 2022	COVID-19; Sept. 2020 wildfires	• Time series plot (of 7–day moving average of normalized sales) • ITSA • Change-point analysis (CPA) • ARIMA with alert thresholds	• ITSA: The start of COVID-19 was associated with a large slope increase in sleep aid sales, suggesting a sustained increase in the use of sleep aids. The wildfires are associated with a small level increase and a slope decrease. • CPA: We found two change points, the first during the initial COVID-19 surge in Los Angeles County in May 2020, after which mean sleep aid sales remained elevated for 18 months; we found no change after the wildfires; a second change point occurred in September 2021, which was not temporally associated with a known pandemic–related event but may suggest the establishment of a new baseline as people adjust to living with COVID-19. This new baseline is still more than twice the pre–pandemic mean, meaning the increase in use of sleep aids is persisting. • ARIMA: We found four periods of anomalous activity in OTC sleep aid sales. These roughly coincided with the period just after each of four large COVID-19 waves in Los Angeles County.

a Adapted from Acosta et al.^19^

Abbreviations: ARIMA, autoregressive integrative moving average; COVID-19, Coronavirus Disease 2019; CPA, change-point analysis; ITSA, interrupted time series analysis; OTC, over-the-counter; PHE, public health emergency; SSRI, selective serotonin reuptake inhibitor.

## Data Availability

Selected data may be made available upon request.
