# Supplementary material for: Integrating Behavioral Health Into Monitoring and Surveillance During Public Health Emergencies: Challenges and Opportunities
Source: Disaster Med Public Health Prep. Author manuscript; Available in PMC 2024 Oct 16. (PMC11483156; doi:10.1017/dmp.2024.127)
Supplement: Supplement 2 [file NIHMS2024047-supplement-Supplement_2.docx]

**Supplement 2: Interview Protocol for Interviews (RQ 2.2)**

**INTRODUCTIONS OF THOSE ON THE CALL**

**CONSENT**

Thank you for taking the time to talk with us today about your perspectives on behavioral health surveillance both prior to and during the nation’s response to COVID-19.

- Does an hour-long conversation still work for you?
- And do we have your permission to record and transcribe this discussion for our internal use only? We won’t link anything you say to you by name or organization.

[Start recording if they agree]

**BACKGROUND ON THE PROJECT**

Our call today is part of a 3-year RAND study, funded by the Centers for Disease Control and Prevention. The slide I’m sharing shows our three key research questions – which I won’t read word by word – but ultimately our goal is to create a practical, user-friendly toolkit to help state, local, tribal, and territorial health departments conduct behavioral health surveillance in the context of disasters – including, but not limited to, COVID-19.

We are focused on community-level impacts of disasters and on early indicators of emotional distress and behavioral health problems that may include subclinical symptoms, risky behaviors, or other early warning signs that public health departments could track and intervene upon during and after public health emergencies. Given that this toolkit is intended to focus more broadly on mental health issues, including upstream prevention efforts needed to mitigate acute needs when disasters occur, the toolkit will not cover how public health departments should use the surveillance information to refer individuals directly for care or to prevent individuals at-risk for suicide from dying. We are also not proposing the creation of new data collection mechanisms, but rather thinking about how we can repurpose existing data to help strengthen behavioral health surveillance.

- Do you have any questions before we get started?

At this stage of the study, we have solicited a lot of input from different informants and have conducted literature reviews, environmental scans, and exploratory data analyses.

Our toolkit will discuss strengths and limitations of more commonly used data sources for public health surveillance like the emergency department visit data from the National Syndromic Surveillance Program. We have also acquired several sources of data that could *potentially* be used for behavioral health surveillance during disasters, that we believe are novel in that they are underutilized for behavioral health surveillance purposes and available at the sub-state level and at least monthly.

Our exploratory data analyses center around the novel data we have acquired for six counties across three states, all of whom have faced shared and unique public health emergencies in the last few years. [share slide with the data sources we selected].

- Have you used or thought about using any of these data in your work previously?
- Are there other data that might be useful for behavioral health surveillance purposes?

We’ll dive into the exploratory data analyses in a few minutes, but first I would love to hear more about your work.

**ORGANIZATION-SPECIFIC QUESTIONS**

- What are you doing in the context of disasters to monitor the behavioral health of the communities you serve? In the context of COVID?
- How is this similar or different from what you did for other public health emergencies that have occurred in your community?
- What kinds of decisions or actions are being informed by these surveillance efforts?
- Are there gaps in understanding or questions/decisions that PH departments are struggling to answer/make due to a lack of behavioral health data? How have you seen/heard that PH departments are using behavioral health data to inform decisions - during/after the pandemic or other disasters?

***[Share slides with exploratory analyses of promising data sources]***

- What do you think is going on/explains these findings? Are they what you would expect or are they surprising?
- What are the cautions/limitations we should be aware of when interpreting these data?

- When in the course of a disaster would it be helpful for health departments to see this data? On what cadence/with what frequency?
- Do you believe your local health department would have the capacity to obtain these data and do these analyses?
  - How might resource constraints influence how often they conduct these analyses and/or share findings?
  - What other factors, like severity of the behavioral health or type of disaster, might influence them as well?

**DATA SOURCE UTILITY/APPLICATIONS/CONSIDERATIONS:**

- We have heard two perspectives: more data or more action. Which resonates with you?
- One of our priorities is to ensure that equity is considered in the context of behavioral health surveillance.
  - What types of surveillance efforts should we prioritize to ensure that the approaches we’re including in the behavioral health surveillance toolkit promote equity?
  - How should we approach challenges of understanding non-representative data? Or imperfect/incomplete data sets?
- Given the lack of behavioral health surveillance systems, we are offering ways to re-purpose existing data. What pitfalls or missteps do we need to be thoughtful to avoid? Any risks or unanticipated consequences we should be considering?
- We are looking for resources to model the toolkit after. Are there any examples of particularly useful toolkits or field guides that you use often?
- What efforts/initiatives are you aware of that we should be sure our work aligns with?
- What federal (or state) initiatives do you see on the horizon that will impact this type of work, help advance it, etc. (e.g., Public Health Data Modernization)
- Are there other data that might be useful for behavioral health surveillance purposes?
- How are you thinking about behavioral health surveillance going forward?

[Wrap-up and inquire about whether they can accept a gift card]
