# Supplementary material for: Integrating Behavioral Health Into Monitoring and Surveillance During Public Health Emergencies: Challenges and Opportunities
Source: Disaster Med Public Health Prep. Author manuscript; Available in PMC 2024 Oct 16. (PMC11483156; doi:10.1017/dmp.2024.127)
Supplement: Supplement 1 [file NIHMS2024047-supplement-Supplement_1.docx]

**Supplement 1: Interview Protocol for Interviews (RQs 1.2-1.3)**

**INTRODUCTIONS**

**(introduce those on the call)**

**CONSENT**

Thank you for taking the time to talk with us today about your [organization’s/state’s/county’s] approach to and experiences with behavioral health surveillance both prior to and during the nation’s response to COVID-19. Our call today is part of a RAND study, funded by the Centers for Disease Control and Prevention, to create guidance that can help state, local, tribal, and territorial health departments conduct behavioral health surveillance. We’ll focus today on COVID-19 but we intend for the guidance to be relevant to a range of public health emergencies, such as natural disasters and terrorist events.

For the purposes of this project we have defined behavioral health as a state of mental health, emotional wellbeing and/or choices and actions that affect wellness. Examples of behavioral health problems include substance abuse, acute psychological distress, suicide and mental illness. This is different from behavioral healthcare, which is an encompassing term including assessment and treatment of mental and/or addictive disorders.

We are seeking to create guidance for conducting timely, community-focused, behavioral health surveillance with a specific focus on early indicators of mental distress and behavioral health problems (e.g., subclinical symptoms, risky behaviors, ‘smoke signals’) during public health emergencies. This guidance will primarily aim to help state, local, tribal, and territorial public health departments to understand behavioral health patterns. For example, to identify the magnitude of behavioral health problems post-disaster, compare post-disaster and pre-disaster patterns, and examine their geographic distribution for hotspots, and examine disproportionate impacts on vulnerable groups. The guidance will not cover how public health departments should use the surveillance information to refer individuals directly for care or to prevent individuals at-risk for suicide from dying.

We will be conducting qualitative interviews lasting 45-60 minutes with state, local, tribal and territorial health departments and with behavioral health providers to get input on how behavioral health surveillance data are used, or could be used, in the wake of a disaster. We are hoping you can provide feedback on:

(1) promising indicators and data sources that could be used for behavioral health surveillance,

(2) possible entities we should speak with for insight on data or methods that could be used for behavioral health surveillance, and

(3) how we could structure guidance on early indicators of disaster behavioral health impacts to make it as useful as possible for these health departments.

Do you have any questions before we get started? And do we have your permission to record and transcribe this discussion for research purposes only? We will keep all comments anonymous.

1. Let’s start with the current pandemic. Is your organization monitoring BH during COVID-19 or any other disasters? [If so]: could you describe your current efforts?

Possible prompts:

- - Would you recommend this type of monitoring to others? Why or why not?
  - How have you used this data? what have you done with it?
  - If, instead of COVID-19, your community experienced a different type of disaster such as a [*wild fire, an earthquake or a tornado--whatever is relevant to their community*] what would be different about the behavioral health data you’d want to collect? What would be similar?

1. What, if any, key decisions are states, health departments, mental health departments, and others making based on behavioral health surveillance data during COVID-19? How could states, local health departments, mental health departments, and others USE behavioral health surveillance data? What would they do with the information?

Possible prompts:

- - What decisions could they make with better behavioral health surveillance data? What would decision makers do with the data?
  - How could the data be used to inform policy or programs?
  - To notify behavioral health providers about possible hot spots of need?
  - To respond to media coverage suggesting there is a cluster of problematic behaviors (e.g., suicide, opioid use) occurring in your community?
  - To inform planning of recovery activities or future preparedness?
  - How has this differed from the way you used this surveillance data in past disasters?

1. Ideally, what behavioral health **indicators** would you recommend state and local public health departments monitor during the COVID-19 response if they wanted to get an early warning of future behavioral health problems in their community? [e.g., homelessness rates, subclinical symptoms of emotional distress, suicidal ideation, use of psychotropic medications]

Possible prompts:

- We’ve been trying to identify what the sweet spot is for public health departments to provide – useful and actionable data. Commonly used mental health indicators (e.g., suicide rates, hospitalizations) do not capture the spectrum of severity and thus do not allow for the prediction of downstream behavioral health needs. What can we do differently? Are there other leading indicators that may predict downstream behavioral health needs?
- We could also probe them about disaster sensitive indicators vs. those that they should be tracking to predict a looming disaster – using our yet to be created conceptual model?
- Why would you recommend these indicators? What are their strengths? Any limitations?
- In reality, based on what data is currently being collected, what indicators would you recommend? Again, what are the strengths and limitations of these?
- How would your recommendations differ for other natural or manmade disasters such as a hurricane, flooding, or terrorist attack?
- What would be the challenges to collecting these indicators? How would you suggest overcoming those challenges?

1. What **data sources** might be useful for behavioral health surveillance at the state or local level during the COVID-19 response?

Possible prompts:

- Are there local organizations affiliated with the Department of Health that might be collecting relevant data? Outside the Department of Health (e.g., local hospital, private providers, insurers)?
- What about sources that would be useful during all-hazards responses? [*Note to interviewer: If needed define what all-hazards means: “integrated approach to emergency preparedness planning that focuses on capacities and capabilities that are critical to preparedness for a full spectrum of emergencies or disasters from natural to manmade disasters.” Centers for Medicare and Medicaid Services*]
- What would be the challenges to using these data sources? How would you suggest overcoming those challenges?

1. Could you tell us about any other entities (e.g., states, public health departments, private organizations) that you’re aware of that are monitoring behavioral health during the COVID-19 response? Or during any other past disasters?

Possible prompts:

- How are they collecting data? How are they analyzing the data? Who can access the data? Who are they providing information to for decision making? Anything else you can tell us about their activities?
- Any other suggestions for who we should speak with during our qualitative interviews?

1. As I mentioned earlier we are working on creating guidance that will help both public health departments to conduct behavioral health surveillance and share information effectively with behavioral health agencies and providers. What should it contain?

Possible prompts:

- - We have been exploring whether it is useful to suggest a specific set of indicators or whether a conceptual model where local indicators and sources of data could be accessed would be more useful. What is the right degree of specificity/standardization for this type of guidance?
  - Are public health departments likely to use this guidance? If not what is a better way to achieve the goal of improving LHD’s use of behavioral health surveillance data?
  - What have you found useful or less useful in the past for similar guidance documents? Any exemplars you would recommend we review?
  - How should it be shared with public health departments and behavioral health providers to inform your COVID-19 response? To inform all-hazards responses?
  - Are there any recommendations for ways that public health and behavioral health providers should work together to improve behavioral health surveillance during COVID-19 response? During future disasters?

Thank you so much again for your time. [Wrap up and discuss next steps].
